# Sulforaphane-induced apoptosis involves the type 1 IP_3_ receptor

**DOI:** 10.18632/oncotarget.8968

**Published:** 2016-08-01

**Authors:** Sona Hudecova, Jana Markova, Veronika Simko, Lucia Csaderova, Tibor Stracina, Marta Sirova, Michaela Fojtu, Eliska Svastova, Paulina Gronesova, Michal Pastorek, Marie Novakova, Dana Cholujova, Juraj Kopacek, Silvia Pastorekova, Jan Sedlak, Olga Krizanova

**Affiliations:** ^1^ Institute of Clinical and Translational Research, Biomedical Research Center, SAS, Bratislava, Slovakia; ^2^ Institute of Virology, Biomedical Research Center, SAS, Bratislava, Slovakia; ^3^ Department of Physiology, Faculty of Medicine, Masaryk University, Brno, Czech Republic; ^4^ Cancer Research Institute, Biomedical Research Center, SAS, Bratislava, Slovakia

**Keywords:** type 1 IP_3_ receptor, sulforaphane, apoptosis, NRF2, nude mice

## Abstract

In this study we show that anti-tumor effect of sulforaphane (SFN) is partially realized through the type 1 inositol 1,4,5-trisphosphate receptor (IP_3_R1). This effect was verified *in vitro* on three different stable cell lines and also *in vivo* on the model of nude mice with developed tumors. Early response (6 hours) of A2780 ovarian carcinoma cells to SFN treatment involves generation of mitochondrial ROS and increased transcription of NRF2 and its downstream regulated genes including heme oxygenase 1, NAD(P)H:quinine oxidoreductase 1, and KLF9. Prolonged SFN treatment (24 hours) upregulated expression of NRF2 and IP_3_R1. SFN induces a time-dependent phosphorylation wave of HSP27. Use of IP_3_R inhibitor Xestospongin C (Xest) attenuates both SFN-induced apoptosis and the level of NRF2 protein expression. In addition, Xest partially attenuates anti-tumor effect of SFN *in vivo*. SFN-induced apoptosis is completely inhibited by silencing of IP_3_R1 gene but only partially blocked by silencing of NRF2; silencing of IP_3_R2 and IP_3_R3 had no effect on these cells. Xest inhibitor does not significantly modify SFN-induced increase in the rapid activity of ARE and AP1 responsive elements. We found that Xest effectively reverses the SFN-dependent increase of nuclear content and decrease of reticular calcium content. In addition, immunofluorescent staining with IP_3_R1 antibody revealed that SFN treatment induces translocation of IP_3_R1 to the nucleus. Our results clearly show that IP_3_R1 is involved in SFN-induced apoptosis through the depletion of reticular calcium and modulation of transcription factors through nuclear calcium up-regulation.

## INTRODUCTION

Sulforaphane (SFN), a dietary isothiocyanate, is a product of myrosinase enzyme hydrolysis of the natural precursor glucoraphanin that occurs in various cruciferous vegetables, including broccoli, Brussels sprouts and cauliflower [1–3]. A variety of effects including anti-inflammatory, antioxidant and anti-tumor properties have been assigned to SFN. It has been observed that intracellular concentrations of isothiocyanates can be 100-200-fold higher than extracellular concentrations [4]. For example, when hepatoma cells are incubated with 100 μM SFN for approximately 30 min, the intracellular concentration reaches approximately 6.4 mM [5]. This gives SFN a potential for therapeutic utilization. We studied sulforaphane, since epidemiological studies suggest that increased dietary intake of cruciferous vegetables is associated with a reduced incidence of cancer [6]. Plasma concentrations of SFN and its metabolites can reach 2.2 and 7.3 μmol/L after consumption of 100 g of standard and high-glucosinolate broccoli, respectively [7]. However, higher concentrations are achieved in tissues and it has been reported that the SFN concentration in the small intestine reached 3 and 13 nmol/g of tissue, which is equivalent to roughly 3–30 μM of total SFN [8]. The plasma levels of isothiocyanates reported so far in the literature (in the μM range, but often <10 μM) were achieved after administration of broccoli preparations (not a pure isothiocyanate preparation), containing doses of isothiocyanate substantially lower than the maximum tolerated ones.

SFN acts through the nuclear factor (erythroid-derived 2)-like 2 (NRF2), which is a critical transcription factor involved in the regulation of cellular defense against oxidative stress. Activation of the Nrf2 by SFN significantly increased the expression of endogenous cytoprotective genes in brain tissue and microvessels. Mice lacking Nrf2 gene have shown that NRF2 regulates not only the inducible expression of numerous cytoprotective genes but also the basal expression of many of these genes.

Postinjury administration of SFN reduced the loss of endothelial cell markers and tight junction proteins and preserved blood-brain barrier function through the activity of Nrf2. Mice lacking the Nrf2 gene did not benefit from the administration of SFN [9]. NRF2 regulates the basal and inducible expression of many antioxidant pathway genes containing antioxidant response elements (AREs) in their transcription control region [10]. SFN has been shown to increase many ARE-dependent antioxidant enzymes in different cell systems [11], such as glutathione S-transferases, glutaredoxin, thioredoxin, and antioxidant enzymes. NRF2 also exerts control over aquaporin, multiple classes of ATPases, and chloride, potassium and calcium channels [12, 13].

Cell death and survival decisions are critically controlled by intracellular calcium homeostasis and dynamics at the endoplasmic reticulum (ER) level. Inositol 1,4,5-trisphosphate receptors (IP_3_Rs) are deeply involved in these processes by mediating calcium flux from the ER into the cytosol [14, 15]. Three types of these channels have been identified – type 1, 2 and 3. Type 1 and 2 IP_3_Rs have been shown to participate in apoptosis induction [16, 17]. Thus, IP_3_Rs may be critical regulators of apoptosis triggered by stimuli that engage ER and/or mitochondrial mechanisms [15, 18, 19]. In our previous work, we showed that apoptosis induction results in an increase of type 1 IP_3_Rs (IP_3_R1) and their subsequent translocation into the nucleus [20]. This translocation could result in a rapid change in calcium concentration status in the nucleus with consequent changes to some responsive elements and transcription factors.

Based on our current knowledge, we propose that SFN may also activate IP_3_Rs and, through these receptors, participate in apoptosis induction. Therefore, the aim of our present study was to determine whether IP_3_Rs are involved in SFN-induced apoptosis.

## RESULTS

**Figure 1:**
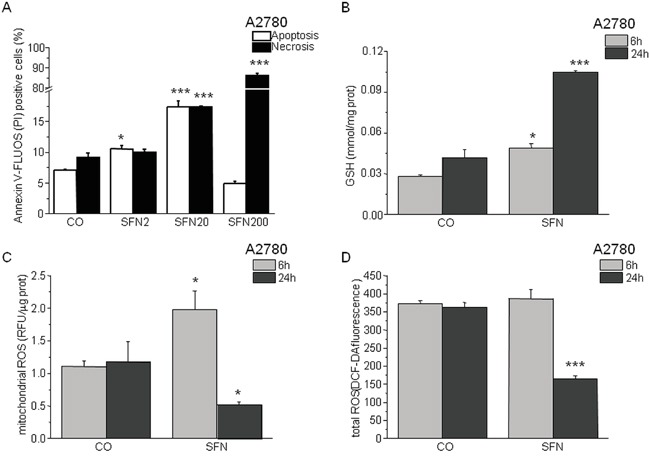
Sulforaphane (SFN) increases apoptosis in a concentration-dependent manner (A) and modulates GSH (B) and ROS (C, D) in a time-dependent manner A2780 cells were treated with SFN in a final concentration 2μM (SFN2), 20μM (SFN20) and 200μM (SFN200) for 24 hours. Apoptosis was measured by Annexin V-FLUOS (*A; empty columns*) and apoptosis/necrosis by propidium iodide (*A; black columns*). SFN2 and SFN20 increased apoptosis significantly, while in SFN200 a rapid necrosis prevails. Due to the SFN treatment, GSH increased in both time intervals *(6h and 24h; B)*, although after the 24 hour's treatment with SFN the GSH increased more significantly. Mitochondrial and total ROS decreased significantly after 24 hours of SFN treatment **C, D.** while after a 6 hour treatment increase in mitochondrial ROS and no change in total ROS was observed. Each column is displayed as mean ± S.E.M and represents an average of three independent cultivations, each performed in triplicates. Statistical significance * compared to corresponding control represents p <0.05 and *** p <0.001.

Flow cytometric analysis of Annexin-V staining confirmed that SFN treatment for 24 hours induces apoptosis in a concentration-dependent manner using low micromolar concentrations, while 200 μM SFN causes massive necrosis in A2780 cells (Figure 1A). Cellular glutathione (GSH) levels (Figure 1B) as well as mitochondrial reactive oxygen species (ROS) production are increased (Figure 1C) after 6 hours of SFN treatment. Both total and mitochondrial ROS levels diminished after 24 hours of SFN treatment, while GSH levels remained increased (Figure 1B, 1C, 1D).

**Figure 2:**
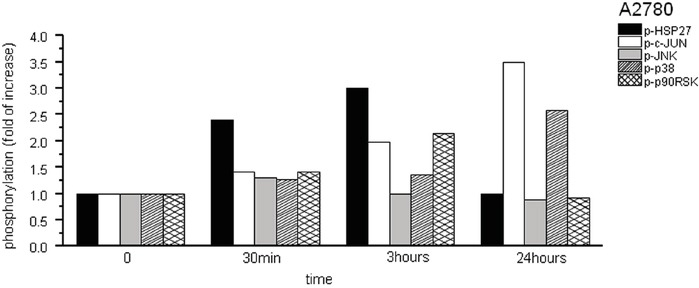
Phosphorylation assay of A2780 cells treated with SFN Due to the SFN (20μM) treatment, phosphorylation of HSP27, cJUN, JNK, p38 and p90^RSK^ proteins was observed from 12 phosphoproteins tested. Phosphorylation was determined in following time periods – 0 min, 30 min, 3h and 24h. Results are displayed as a fold of increase compared to corresponding controls.

Within 30 min of SFN (20 μM) treatment, we observed a 2.4-fold increase of HSP27 phosphorylation as well as a slight (approximately 40%) increase in JNK, MEK1, p38, p90^RSK^, and c-JUN phosphorylation above the control sample levels (Figure 2). No significant change in phosphorylation was observed in the 7 other proteins tested (data not shown). Phosphorylation of p90^RSK^, c-JUN, and HSP27 peaked between 30 min and 3 hours of SFN treatment. The phosphorylation of c-JUN further increased after 24 hours of treatment (3.5-fold), as well as p38 (2.5-fold) in comparison to corresponding control cells. A sign of increased mitotic cell proportion – the phosphorylation of histone H3 – was observed as well (data not shown). Phosphorylation of histone H3 on Ser-10 is regarded as an epigenetic mitotic marker and is tightly correlated with chromosome condensation during both mitosis and meiosis. However, it was also reported that histone H3 Ser-10 phosphorylation occurs when cells are exposed to various death stimuli, suggesting a potential role in the regulation of apoptosis [21].

**Figure 3:**
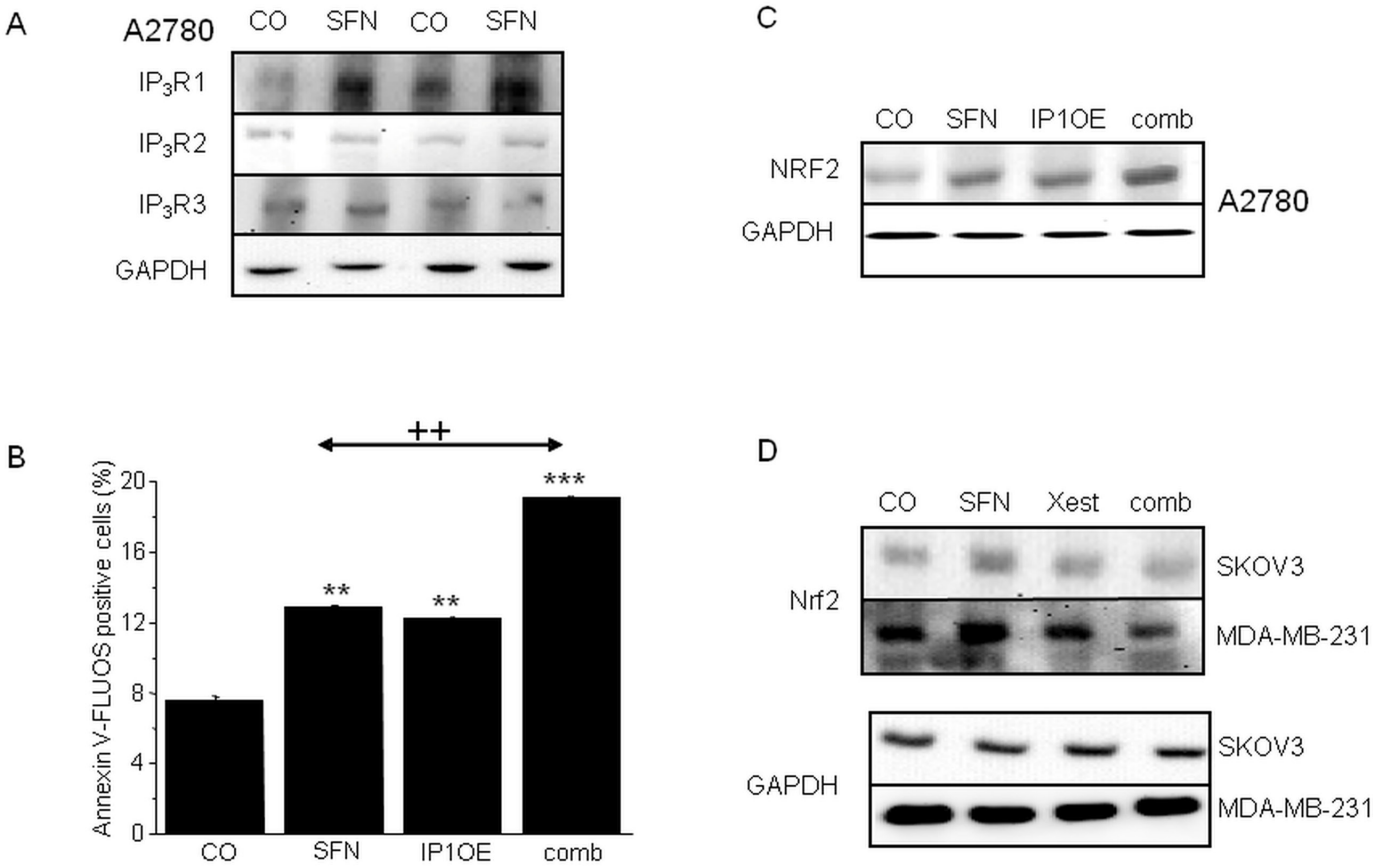
IP3Rs are involved in increased NRF2 expression due to SFN (20μM) treatment in A2780, SKOV3 and MDA-MB-231 cells SFN increases IP_3_R1, but not IP_3_R2 and IP_3_R3 protein amount **A.** in 10^4^ and 10^5^ A2780 cells, respectively. As a loading control, glyceraldehyde-3-phosphate dehydrogenase (GAPDH) was used. Further, we transfected cells with the IP_3_R1 (IP1OE) and determine NRF2 protein and the amount of apoptotic cells after a subsequent SFN treatment **B, C.** We observed significant increase in both, NRF2 protein (C) and apoptosis (B) compared to wild A2780 cells treated with SFN. Also, SFN-induced increase of NRF2 protein was observed in SKOV3 and MDA-MB-231 cells. This increase was prevented by parallel treatment with Xest and SFN **D.** Each column is displayed as mean ± S.E.M and represents an average of three independent cultivations, each performed in triplicates. Statistical significance ** compared to control represents p <0.01 and *** p <0.001. Statistical significance ++ compared to SFN treated cells represent p <0.01.

In addition, Western blot analysis confirmed the SFN-induced increase of IP_3_R1 expression, in contrast to IP_3_R2 or IP_3_R3 receptors, which were not affected by the treatment (Figure 3A). Moreover, a rise in NFR2 protein expression was observed in cells that overexpressed IP_3_R1 (IP1OE; Figure 3B) compared to control A2780 cells. When IP1OE cells were treated with SFN (comb; Figure 3B), a further increase of NRF2 protein level compared to control untreated cells was observed. Interestingly, IP1OE cells exhibit a higher percent of apoptotic cells compared to control A2780 cells and also exhibit an additional increase in SFN-induced apoptosis when compared to SFN-treated A2780 cells (Figure 3C). Involvement of the IP_3_Rs on NRF2 expression was verified by parallel treatment of SFN and Xest in two other cell lines – SKOV3 and MDA-MB-231 (Figure 3D). When this combination was used, no increase in NRF2 was visible compared to SFN-treated group (Figure 3D).

**Figure 4:**
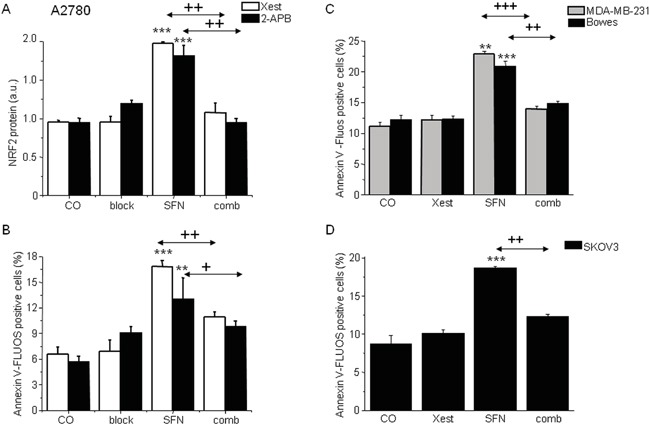
NRF2 protein (A) and apoptosis induction (B, C, D) in the cells treated with sulforaphane (SFN), IP3R blockers (block), or combination of both, SFN and blocker (comb) To show the effect of the IP_3_R blockers, experiment with a less specific 2-aminoethoxydiphenyl borate (2-APB; black columns) or experiment with the more specific Xestospongin C (Xest; empty columns) was performed on A2780 cells **A, B.** Increase in the amount of apoptotic cells due to SFN treatment (20μM) was observed also in MDA-MB-231 cells (*C*; gray columns), Bowes cells (*C*; black columns) and SKOV3 cells (*D*, black columns). When all these cells were treated in parallel with SFN and Xest (comb), no increase compared to controls was observed **B, C, D.** Each column is displayed as mean ± S.E.M and represents an average of at least three independent cultivations, each performed in triplicates. Statistical significance ** compared to corresponding control represents p <0.01 and *** p <0.001. Statistical significance + compared to SFN treated group represents p <0.05, ++ p <0.01 and +++ p <0.001.

In ovarian A2780 cells, SFN treatment (20 μM) for 24 h significantly increases the expression of NRF2 protein (Figure 4A), markers of ER stress CHOP and ATF4 (data not shown), and the fraction of apoptotic cells (Figure 4B). The increase in these parameters was almost completely abolished when cells were treated with SFN together with an IP_3_R blocker (Figure 4A, 4B block) – a non-specific IP_3_R blocker 2-APB (Figure 4A, 4B black columns) or a more specific IP_3_R inhibitor Xest (Figure 4A, 4B, empty columns). To test if the inhibitory effect of Xest on the SFN-induced increase of NRF2 protein and apoptosis is a common phenomenon in tumor cells, we performed these experiments using another ovarian tumor cell line SKOV3 and the breast cancer cell line MDA-MB-231. Our results suggest the involvement of IP_3_R in the SFN-induced increase of NRF2 protein and apoptosis similar to what we have found in the A2780 cell line (Figure 4C, 4D).

**Figure 5:**
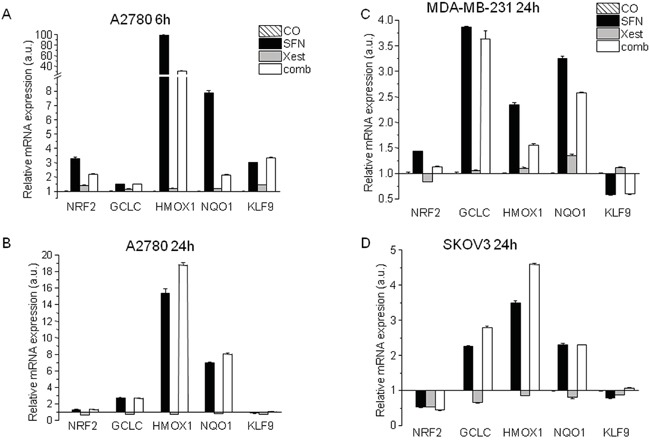
Changes in mRNA levels of NRF2 and the downstream genes GCLC, HMOX1, NQO1, and KLF9, after 6 and 24 hours of SFN treatment Relative mRNA expression of NRF2, GCLC, HMOX1, NQO-1 and KLF9 genes determined in A2780 untreated cells or cells exposed to 20 μM SFN, 1 μM Xest and combination of 20 μM SFN; 1 μM Xest for 6 hours **A.** and 24 hours **B.** Relative mRNA expression of the same genes was determined also in MDA-MB-231 cells **C.** and SKOV3 cells **D.** in untreated cells or cells exposed to 20 μM SFN, 1 μM Xest and combination of 20 μM SFN; 1 μM Xest for 24 hours. Total RNA was isolated and real time qRT-PCR was performed as described in Material and Methods. The data are expressed as the means ± SEM. β-actin was used as a reference gene.

Because SFN affects ROS production in a time-dependent manner (Figure 1B, 1C), we measured changes in mRNA levels of NRF2 and the downstream genes GCLC, HMOX1, NQO1, and KLF9, after 6 and 24 hours of SFN treatment (Figure 5). In A2780 cells, the early (6 hours) increase in NRF2 mRNA was not observed at the 24-h time point. Nevertheless, an increase in NRF2-regulated downstream genes GCLC, HMOX1, and NQO-1 was found in SFN- and SFN-Xest-treated cells after 24 h of treatment. In contrast, the initial SFN-induced increase of KLF9 mRNA was reversed to a down-regulation after 24 h of treatment (Figure 5B), an effect also observed in SKOV3 and MDA-MB-231 cells (Figure 5C, 5D). This result may suggest a common mode of redox regulations in different types of carcinoma cell lines.

**Figure 6:**
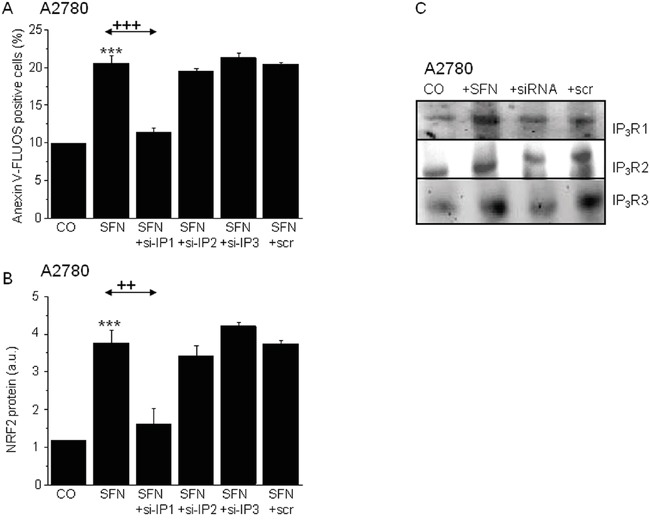
Involvement of the IP3R1, but not IP3R2 or IP3R3 in the SFN (20μM) induced apoptosis (A) and NRF2 protein increase (B) Silencing of the IP_3_R1 and subsequent treatment with SFN (SFN+si-IP1) prevented SFN induced apoptosis **A.** and also SFN induced NRF2 protein increase **B.** Silencing of the IP_3_R2 (SFN+si-IP2) and IP_3_R3 (SFN+si-IP3) does not reveal any changes neither in SFN induced apoptosis (A), nor in the NRF2 protein increase (B). Efficiency of the silencing was verified by Western blot analysis with appropriate antibodies. The gels proved the silencing of the IP_3_R1, IP_3_R2 and IP_3_R3 **C.** Each column is displayed as mean ± S.E.M and represents an average of at least two independent cultivations, each performed in triplicates. Statistical significance ** compared to corresponding control represents p <0.01 and *** p<0.001. Statistical significance ++ compared to SFN treated group represents p <0.01 and +++ - p<0.001.

To more deeply analyze the involvement of IP_3_Rs in SNF-induced apoptosis, we selectively silenced mRNA of type 1, 2 and 3 IP_3_Rs in A2780 cells to differentiate which type of IP_3_R is responsible for SFN-induced changes (Figure 6). Silencing of IP_3_R1 gene expression completely prevented the SFN-induced increase in apoptosis and NRF2 protein expression (Figure 6A, 6B), while no effect was observed when IP_3_R2 or IP_3_R3 were silenced, compared to a loading control.

**Figure 7:**
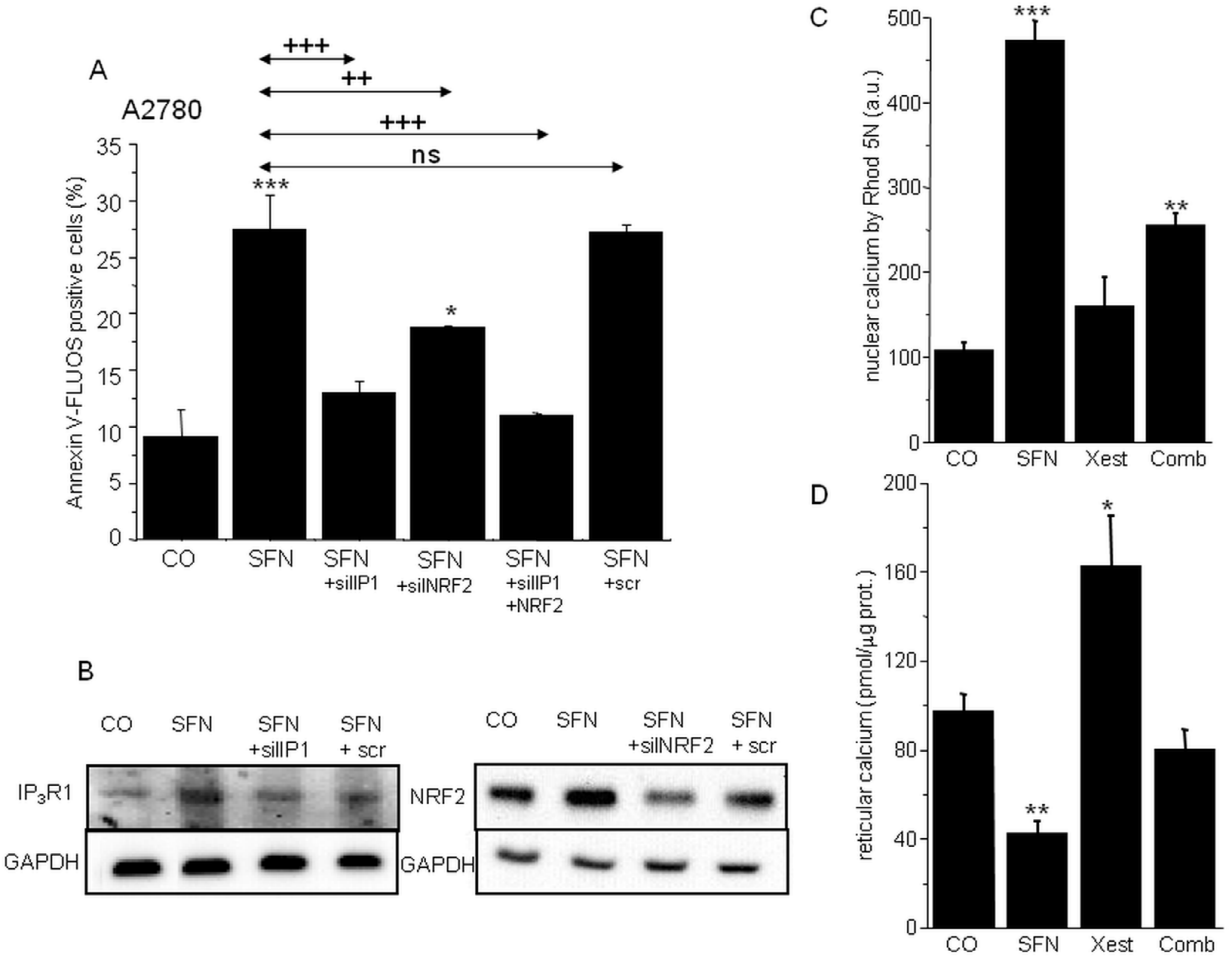
Apoptosis determination in samples with silenced either IP3R1 or NRF2, and combination of both (A) clearly revealed that both these proteins are responsible for the SFN-induced apoptosis Silenced IP_3_R1 and NRF2 revealed significantly lower protein levels **B.** Housekeeper GAPDH was used as a control (B). SFN elevates calcium content in the nuclei C. while depletes calcium from the endoplasmic reticulum D. Combined treatment of the SFN and IP_3_R blocker Xestospongin C reverses these effects **C, D.** Each column is displayed as mean ± S.E.M and represents an average of at least one **A.** or two (C, D) independent cultivations, each performed in triplicates. Statistical significance * compared to corresponding control represents p <0.05, ** p <0.01 and *** p <0.001. Statistical significance ++ compared to SFN and/or SFN+scr treated group represents p <0.01 and +++ p<0.001.

To determine the possible mutual involvement of IP_3_R1 and NRF2 in SFN-induced apoptosis, the IP_3_R1 and NRF2 genes were silenced either separately or in combination in cells subsequently treated with SFN (Figure 7A). IP_3_R1 silencing completely prevented SFN-induced apoptosis, while NRF2 silencing decreased SFN-induced apoptosis nearly 45%. Combined silencing of the IP_3_R1 and NRF2 mRNA resulted in preventing SFN-induced apoptosis. The average efficiency of IP_3_R1 silencing was more than 65% and for NRF2 was more than 60%, as determined by Western blot analysis (Figure 7B).

**Figure 8:**
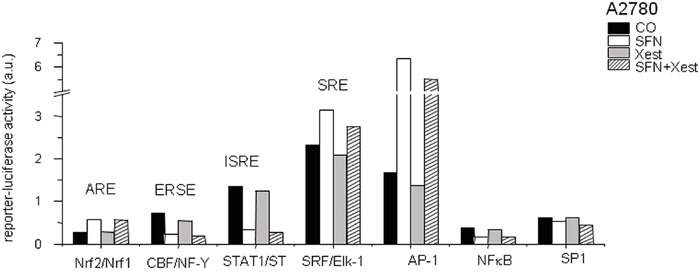
Cignal reporter assay of A2780 cells treated with SFN Cignal reporter assay showing regulation of some responsive elements due to the SFN (20μM) treatment. Increase up to 0.02-fold in the reporter-luciferase activity was considered to be statistically non-significant.

Because IP_3_Rs are calcium-release channels that release calcium from the intracellular stores, we measured nuclear and reticular calcium content. SFN treatment for 24 hours elevated nuclear calcium levels, but this increase was partially abolished by treatment with the IP_3_R blocker Xest (Figure 7C). In contrast, treatment with SFN decreased reticular calcium, and this effect was partially reversed by Xest (Figure 7D). To find the effect of calcium on transcription factors, we compared the activity of 45 different signaling pathways in control A2780 cells and SFN- and SFN with Xest-treated cells. Using pathway-focused transcription factor-responsive luciferase constructs, we observed a significant increase in the MAPK and antioxidant response pathways (AP1, SRE, and ARE promoter sequences) and a decrease in endoplasmic reticulum stress (ERSE) and type I interferon regulation pathways (ISRE promoter) in SFN-treated cells compared to controls (Figure 8). Interestingly, the activity of transcription factor-responsive constructs was similar in both Xest- and SFN+Xest-treated cells when compared to control and SFN-treated cells. We have observed non-significantly diminished activity of SRE and AP-1 in SFN+Xest versus SFN-treated cells.

**Figure 9:**
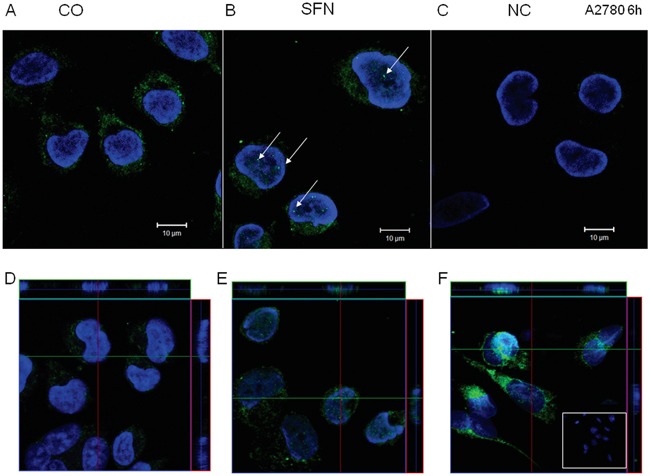
IP3R1 immunofluorescence of control (A, D) and SFN-treated (B, E) A2780 cells IP_3_R1 was localized in the endoplasmic reticulum in control cells and partially translocates to the nuclei in SFN-treated cells (B; white arrows). In order to show that IP_3_R1 are really in the nuclei, we performed z-stacks from control **D.** and SFN-treated **E.** cells. Orthogonal views **D, E, F.** give a middle plane of z-stacks crossing the nuclei and also xz and yz sections through the entire thickness of cells along green and red lines, clearly showing presence of the IP_3_R1 signal (E) and also signal for ER marker - calnexin **F.** within nuclei in SFN treated cells. Signal was not observed in control, non-treated cells (D). As a negative control (C), cells were treated only with the secondary antibody. Nuclei stained by DAPI are shown in blue.

Compared to control untreated cells (Figure 9A), immunofluorescent labeling of IP_3_R1 clearly shows the translocation of this receptor into the nucleus (Figure 9B). A negative control, where the IP_3_R1 antibody was omitted, did not have any signal (Figure 9C). Translocation of IP_3_R1 into the nucleus due to SFN treatment was verified by confocal microscopy and subsequent z-stacks of control (Figure 9D) and SFN-treated cells (Figure 9E). Calnexin, a typical ER marker, was translocated to the nucleus due to SFN treatment as well (Figure 9F), suggesting that the part of the ER enriched in IP_3_R1 moved to the nucleoplasm.

**Figure 10:**
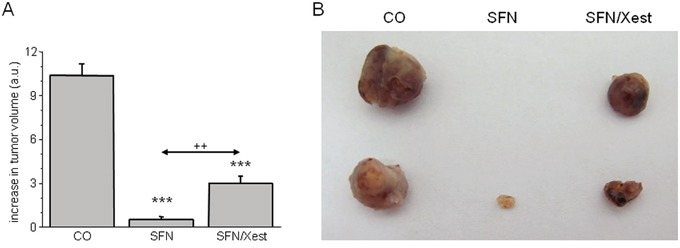
Differences in tumor size in response to SFN and SFN/Xest treatment Nude mice developed tumors after s.c. injection of A2780 cells. After 7-days treatment with SFN, increase in tumor's volume was significantly lower compared to untreated controls **A.** When mice were treated with SFN and IP_3_R blocker Xest, volume of the tumors was lower than in untreated mice, but higher that in SFN treated mice only **A.** Each column represents mean ± S.E.M. and is an average of 3 mice. Statistical significance *** represents p < 0.001 compared to untreated controls and ++ represents p < 0.01 compared to SFN treated group. Tumors from control, SFN and SFN/Xest treated mice are shown in part B.

Involvement of IP_3_R1 was also tested *in vivo* on a model of nude mice with developed tumors after injecting A2780 cells. Mice were treated either with SFN alone, or with SFN in combination with Xest. SFN treatment for 7 days significantly reduced growth of the tumor (Figure 10). When animals were treated with SFN and Xest, decrease in tumor growth was not so pronounced than in SFN-treated mice (Figure 10A, 10B).

## DISCUSSION

Isothiocyanates have been shown to have substantial chemopreventive activity against various human malignancies due to their ability to induce apoptosis [3, 22]. Although there are many causes for apoptosis induction, upstream signaling mechanisms have not yet been fully defined. Our study confirmed that sulforaphane, a naturally occurring isothiocyanate derived from cruciferous vegetables, induces apoptosis in a concentration dependent manner in ovarian and breast carcinoma cell lines [23, 24]. We demonstrate for the first time that IP_3_R1 plays a crucial role in the mechanism of SFN-induced apoptosis.

We observed, as an early effect, a massive phosphorylation of HSP27 in SFN-treated ovarian A2780 cells, which completely disappears after 24 h of SFN treatment. It has already been shown that sustained HSP27 phosphorylation leads to its nuclear sequestration and the subsequent dissociation of its large oligomers and a decrease in its chaperone activity, thereby further compromising the death inhibitory activity of HSP27 [25]. Under stress conditions, HSPs are dispatched to correct conformation and repair misfolded proteins. Although elevated expression levels of HSPs in malignant cells are considered cytoprotective by preventing apoptosis [26, 27], another regulatory system that removes proteins that are beyond repair, the proteasome, could be activated by SFN [28]. Based on our result, we speculate that the phosphorylation of HSP27 in the early phase of the SFN treatment might be an attempt to disrupt cancer cell viability.

SNF is a potent inducer of the KEAP1/NRF2 pathway and acts through the antioxidant response element (ARE) that protects against GSH depletion and oxidation and attenuates inflammation by inhibiting the NFκB pathway [29, 30]. Although SFN has been shown to increase levels of ROS [31, 32], we have shown time-dependent changes in ROS levels. At an early stage, SFN increases mitochondrial ROS levels in A2780, but after 24 hours, mitochondrial ROS levels are abolished due to activation of the NRF2 and ARE-induced antioxidant response. Activation of the transcription factor NRF2, a master response regulator of oxidative stress, is induced by many chemopreventive compounds [33, 34]. NRF2 mRNA was increased after 6 h of SFN treatment in A2780 cells but after 24 h was decreased in SKOV3 cells, even though a small increase was still observed in A2780 and MDA-MB-231 breast carcinoma cells. Recently, a new mode of regulation for the KEAP1-NRF2 pathway balance through NRF2 stabilization has been suggested [35]. In our study, the increased NRF2 protein expression after 24 hours of treatment was diminished using both a nonspecific (2-APB) and a specific IP_3_R blocker, Xest. In addition, both inhibitors decreased the proportion of SFN-induced apoptotic cells. These results suggest that IP_3_Rs are involved in SFN-induced apoptosis, possibly through the modulation of NRF2 stability. This resembles situations where the suppression of calcium release from IP_3_R-sensitive stores mediates anti-apoptotic effects and the absence of IP_3_R-mediated calcium signaling supports the development of aggressive T-cell malignancies [36, 37].

IP_3_Rs are intracellular calcium channels that are emerging as key sites for the regulation of pro- and anti-apoptotic factors [38]. In addition to the direct role of IP_3_Rs in the initiation of apoptosis by providing a conduit for endoplasmic reticulum to mitochondria calcium transfer, there are several additional feedback mechanisms that have been proposed that allow IP_3_Rs to play a role in amplifying calcium-dependent apoptotic pathways [39]. Until now, the involvement of IP_3_Rs in the process of apoptosis has been mainly ascribed to IP_3_R1 [17, 40, 41] and IP_3_R2 [16, 42]. In this study, we have shown that type 1 IP_3_Rs are involved in SFN-induced apoptosis because its silencing prevented SFN-induced apoptosis. Silencing of the type 2 and 3 IP_3_Rs does not have any effect on SFN-induced apoptosis. In addition, silencing of IP_3_R1 prevented the SFN-induced increase in NRF2 protein expression. Recently, it was shown that the activation of NRF2 with quercetin or oxidative stress reduced the expression of IP_3_R3 and calcium signaling in normal cholangiocytes [43]. In our study, the overexpression of IP_3_R1 and subsequent SFN treatment rapidly increased apoptosis in ovarian A2780 cells as well as levels of NRF2 protein. Based on these results, IP_3_R1 affects the promoter activity of genes, including NRF2, through an increase in nuclear calcium. It has been shown that SFN induces the nuclear translocation of NFκB, whose promoter function is regulated by the redox chaperone activity of APE-1/REF-1 [30, 44]. Our data reveal that the status of the nuclear redox milieu is further superimposed by the time-dependent modulation of KLF9, a recently described amplifier of oxidative stress [45].

Using a luciferase reporter system, we have shown that SFN significantly increases AP1 and SRE reporter activity, acting through AP1 and Elk-1/SRF transcription factor binding, respectively. We observed a marked increase in activation of c-JUN, a dimeric partner of immediate-early gene FOS, in accordance with the functioning of AP1. The binding of serum response factor (SRF) to SRE recruits accessory factors such as the Ets-family ternary complex factors (TCFs) Elk-1, Elk4/SAP1 and Elk3/SAP3 [46]. The early phosphorylation of P90^RSK^, a downstream target of ERKs, and the increase in nuclear calcium are in line with requirements for SRF activation [47]. Moreover, a further increase in SRF function could be expected due to the activation of p38 MAPK via its downstream target MAPKAP-K2 (MK2) [48]. Indeed, the early activation of JNK, p38, and P90^RSK^ may support the involvement of the SRF transcription factor in cell response to SFN treatment. Previous studies have shown the ability of the SRE-specific transactivators Elk-1 and SRF to bind N-terminal transactivation, bromo, acetyltransferase, or C-terminal transactivation domains of p300/CBP, even in the absence of a protein phosphorylation [49]. We speculate that due to the inhibition of HDAC [50], SFN might play a role in p300/CBP regulation of Elk-1 and SRF transcriptional activity. Recent studies have revealed SFN-induced inhibition of both STAT1 phosphorylation as well as IFNγ and TNFα-induced NFκB activation through the induction of HO-1 [51] that correspond to the decreased ISRE and NFκB activities observed in our study. It is reasonable to assume that IRSE activity is the result of a phospho-acetyl balance switched to the side of STAT1 acetylation that counteracts IFN-induced STAT1 phosphorylation [44]. Moreover, HO-1, a downstream target of NRF2 activity plays a protective role against ER stress through PERK-supported trafficking of NRF2 into the nucleus [52]. Similarly decreased NFκB activity by SFN was found in breast and prostate carcinoma cells as well [44, 53].

As previously mentioned, transcription factor activities are modulated by changes in nuclear calcium. In our previous work [20], we showed that IP_3_R1 in the cell nucleus is involved in the early process of apoptosis by causing IP_3_R1 containing cluster formations, most likely by fusion of the nucleoplasmic reticulum and translocation of the IP_3_R1 to the nucleus. The current characterization of a nucleoplasmic-reticular network as an IP_3_-sensitive calcium store that traverses deep within the nucleus has far-reaching implications in our understanding of intricate nuclear events that critically depend on the appropriate regulation of nuclear calcium signals in both health and disease [54].

Role of the IP_3_Rs in tumor suppressor effect of SFN was studied also *in vivo* on athymic nude mice with subcutaneous tumors induced by injection of A2780 cells. After SFN treatment, tumor's growth was suppressed and the volume of a tumor was much lower compared to controls. When SFN treatment occurred in the presence of IP_3_R blocker – Xest, tumor volume was higher compared to sole SFN treatment, but still lower than in untreated mice. This observation supports the *in vitro* results that part of the tumor suppressor effect includes IP3 receptors.

In summary, we have shown that type 1 IP_3_R, but not type 2 and 3, participates in the SFN-induced apoptosis of carcinoma cell lines, most likely through the changes in nuclear calcium levels due to IP_3_R1 translocation from the ER to the nucleus. Involvement of IP_3_Rs in SFN induced apoptosis was supported by results from *in vivo* experiments.

## MATERIALS AND METHODS

### Cells and treatment

Most experiments were performed on ovarian carcinoma A2780 cells, although some of the crucial experiments were verified on SKOV3 and MDA-MB 231 cells. The A2780 human ovarian cancer cell line was established from tumor tissue from an untreated patient. These cells were incubated in RPMI 1640 medium (Sigma Aldrich, USA), supplemented with 2% glutamine, 10% fetal bovine serum (Sigma Aldrich, USA) and penicillin/streptomycin antibiotics (both from Calbiochem, Merck Biosciences, Darmstadt, Germany). To verify the effect of SFN, some experiments were performed in parallel on the human ovarian carcinoma cell line (SKOV3) and breast cancer cell line (MDA-MB 231), which were cultivated under same conditions as A2780 cells. All cells were cultured in a humidified atmosphere at 37°C and 5% CO_2_. After plating, cells were treated for 24 hours with SFN (Calbiochem, Merck Biosciences, Darmstadt, Germany) in a final concentration 2, 20 or 200 μM (mostly in a final concentration 20 μM). As inhibitors of the IP_3_ receptors, 10 μM 2-aminoethoxydiphenyl borate (2-APB) and 1 μM Xestospongin C (Xest; Sigma Aldrich, USA) were used.

### Phosphorylation assay

Multiplex evaluation of phosphorylated levels of the proteins of interest was performed with the xMAP Luminex platform (Luminex, Austin, TX), which combines the principle of a “sandwich” immunoassay with fluorescent bead–based technology for analysis of up to 100 different analytes in a single microtiter well in 96-well plates. A panel of 13 phosphoproteins: phosphorylated AKT (Ser473), c-JUN (Ser63), ERK1/2 (Thr202/Tyr204, Thr185/Tyr187), GSK-3α/β (Ser21/Ser9), histone H3 (Ser10), HSP27 (Ser78), IGF-1R (Tyr1131), IκBα (Ser32/Ser36), JNK (Thr183/Tyr185), MEK1 (Ser217/Ser221), p38 MAPK (Thr180/Tyr182), p70 S6 kinase (Thr421/Ser424), P90^RSK^ (Thr359/Ser363), were analyzed in a 96-well format using the Bio-Plex suspension array system, according to manufacturer' instructions (Bio-Rad Laboratories, Hercules, CA).

### Intracellular and mitochondrial reactive oxygen species (ROS) assay

For this assay, mitochondrial fraction from the A2780 cells was isolated. Mitochondrial ROS production was measured by modified method of [55] by a fluorimetric method using the probe 2′,7′-dichlorodihydrofluorescein diacetate (DCFH2-DA). Crude mitochondrial pellet was resuspended in 100 μl of phosphate saline buffer (pH 7.4) with 1% TWEEN. Samples were diluted into 300 μl of freshly prepared 0.1 M KH_2_PO4 buffered to pH 7.4 with KOH and 1 mM solution of DCFH2-DA in DMSO. As a blank sample for auto-oxidative process, well with buffers and DCFH2-DA was measured in parallel. Fluorescence was excited at 489 nm and measured at 525 nm on the fluorescence scanner BioTek (BioTek, Germany) at 37°C for 30 minutes.

### Glutathione assay

Glutathione (GSH) content in the cells was measured by Glutathione assay kit (Calbiochem, Germany) according to manufacturer's manual. The assay is based on substitution of thioethers between patented reagent and all mercaptans presented in the sample. In the second step, formation of chromophoric thione under alkaline conditions occurs, which is measured at 400 nm. As a positive control, cells treated for 24 hours with 40 μM pyocyanine (Sigma Aldrich, USA) were used. Results were expressed as mmols of GSH/mg of protein in sample.

### Detection of apoptosis with Annexin-V-FLUOS

After the SFN treatment, A2780 cells were gently scraped and pelleted at 100 x g for 5 min. Cells were then washed with 1 ml of PBS (phosphate buffered saline pH 7.4) and cell pellet was resuspended in 200 μl of Annexin-V-FLUOS/propidium iodide labeling solution (Roche Diagnostics.USA) and incubated at room temperature in dark for 20 minutes according the manufacturer's protocol. After the incubation, samples were placed on ice and measured on BD FACSCanto II flow cytometer (Becton Dickinson, Ann Arbor, USA).

### Western blot analysis

Cells were scraped and suspended in 10 mM Tris-HCl, pH 7.5, 1 mM phenylmethyl sulfonylfluoride (SERVA, Germany), protease inhibitor cocktail tablets (Complete EDTA-free, Roche Diagnostics, Germany) and subjected to the centrifugation for 10 min at 10 000 x g at 4°C. The pellet was resuspended in Tris-buffer containing 50 μM 3-[(3-Cholamidopropyl)dimethyl-ammonio] 1-propanesulfonate (CHAPS; Sigma, USA), and then incubated for 10 min at 4°C. The lysate was centrifuged for 10 min at 10 000 x g at 4°C. Protein concentration of supernatants was determined by the method of Lowry [56]. Twenty to sixty μg of protein extract from each sample was separated by electrophoresis on 10% SDS polyacrylamide gels and proteins were transferred to the Hybond-P membrane using semidry blotting (Owl, Inc., USA). Membranes were blocked in 5% non-fat dry milk in Tris-buffered saline with Tween 20 (TBS-T) for overnight at 4°C and then incubated for 1 hour with IP_3_R1 rabbit polyclonal antibody (1:1000 dilution, Sigma Aldrich, USA), or NRF2 mouse monoclonal antibody (1:1000 dilution, Sigma Aldrich, USA), a rabbit polyclonal antibody to IP_3_R2 (1:1000 dilution, Chemicon, USA) and rabbit polyclonal to IP_3_R3 (1:1000 dilution, Abcam, Cambridge, UK). For CREB-2 binding protein staining, a rabbit polyclonal antibody raised against a peptide mapping near the C-terminus of ATF4 of human origin was used (1:1000 dilution, Santa Cruz Biotechnology, CA, USA). GADD 153 (1:1000 dilution, Santa Cruz Biotechnology, CA, USA) rabbit polyclonal antibody was used against CHOP. Following washing, membranes were incubated with secondary antibodies to rabbit and mouse IgG conjugated to horseradish peroxidase (1:10 000 dilution, GE Life Sciences, USA) for 1 hour at room temperature. For relative quantification, each membrane was re-probed for housekeeper GAPDH mouse monoclonal antibody (1:5000 dilution, Abcam, Cambridge, UK). An enhanced chemiluminiscence detection system (ECL Plus, Amersham Biosciences) was used to detect bound antibody. The optical density of individual bands was quantified using PCBAS 2.0 software.

### Transient transfection

A2780 cells were plated onto 60-mm Petri dishes to reach approximately 70% monolayer density on the following day. Transfection was performed with 6 μg of plasmid DNA using Turbofect Transfection Reagent (Fermentas, Thermo Fisher Scientific) according to the manufacturer. After 24 hours, the transfected cells were trypsinized, plated onto 6-well plates, allowed to attach and treated with SFN additional 24 hours. Cells were harvested for FACS analysis and protein extracts at the end of treatment period.

### Silencing by siRNAs

Cells were grown to density of 4 × 10^5^ in 6-well plates in the RPMI medium with 10% FBS. For silencing procedure SMART POOL siRNAs from Dharmacon (Thermo Fisher Scientific, USA) were used. Cells were washed with RPMI medium without FBS and 100 pM of each SMART pool ON-TARGET plus siRNA mixed with Dharmafect 1 transfection reagent and RPMI were applied to the cells in total volume of 1 ml. After 4 hours, RPMI medium to 3 ml per well and FBS to 10% were added, in order to avoid cytotoxicity. After 24 hours, silencing procedure was repeated together with treatment with SFN and cells were grown additional 24 hours in serum free medium. In this way were mRNAs silenced for 48 hours and treatment with drugs was 24 hours in length. siRNAs used were as follows: SMARTpool ON-TARGETplus ITPR1 siRNA (Dharmacon, L-006207-00-0005), SMARTpool ON-TARGETplus ITPR2 siRNA (Dharmacon, L-006208-02-0005), SMARTpool ON-TARGETplus ITPR3 siRNA (Dharmacon, L-006209-00-0005), SMARTpool ON-TARGETplus NFE2L2 siRNA (Dharmacon, L-003755-00-0005). As a negative control (scrambled), ON-TARGETplus NON-targeting Pool siRNAs were used (Dharmacon, USA).

### [Ca^2+^]free measurement with Rhod −5N

A2780 cells were plated at density 2 × 10^6^ in 75 cm^2^ bottom flasks, 24 hours prior to the treatment. Twenty four hours after treatment, cells were scraped from flasks, sedimented, washed with PBS and gently lysed with 500 μl of cell lysis buffer for cytoplasmic and nuclear protein isolation (ProteoJet™ Fermentas, Thermo Fisher Scientific, USA) with DTT added to the final concentration of 1 mM. Isolation of cell nuclei was performed according to the kit producer. Reticular fraction was isolated according to [17]. Pellets from nuclear and reticular fractions were homogenized in 200 μl of nuclear lysis buffer from ProteoJet™ kit and pipetted to wells in 24 well plate. To each sample Rhod −5N fluorescent dye was added to the final concentration of 20 μM. Measurements were performed on the BioTec fluorescent reader (BioTec, Germany) at 551 nm (excitation) and 576 nm (emission). After measuring of fluorescence (F), signal was quenched by adding of EGTA solution with pH 7.0 to the final concentration of 0.25 mM, 1.0 mM and 2.5 mM and 5.0 mM. Signal was saturated at 2.5 mM EGTA (Fmin). Fmax value was measured by adding of 100 mmol/l CaCl_2_ to the final concentration of 0.5 mM. Final values of [Ca^2+^]_free_ were calculated according to the formula: [Ca^2+^]_free_ = Kd [(Fmax − F) / (F − Fmin)] where Kd for Rhodamine −5N is 320 μM.

### Cignal finder reporter array (SABiosciences)

Cignal Finder Reporter Array consists of 45 dual-luciferase reporter assays enabling rapid and reliable identification of signaling pathway activities and to determine the effects of various treatments. Cell-based Multi-Pathway Activity Assay was performed in A2780 cell line (6 × 10^4^ cells/well) according to instructions of the manufacturer. Transfection was performed using Attractene Transfection Reagent (0.3 μl/well, Qiagen). Twenty four hours post transfection, medium was replaced with fresh one, containing SFN (20 μM), Xest (1 μM), combination of both or DMSO only, which served as a controland cells were treated for additional 24 hours. The activity of each signaling pathway was analyzed using Dual Luciferase Reporter Assay Kit (Promega, USA) and measured using Biotek Synergy HT luminometer. Results were calculated for each transfectant and analyzed by the Data Analysis Software (SABiosciences, USA). The change in the activity of each signaling pathway was determined by comparing the normalized luciferase activities of the reporter in SFN/Xest treated versus SFN treated transfectants.

### Analysis of the gene expression by real-time PCR

Cell lines were treated with SFN (20 μM), Xest (1 μM), and combination of both for time as indicated. Total RNA was isolated using TRIzol® Reagent (Ambion®, USA). Two micrograms of total RNA was reverse transcribed with RevertAid™ H minus First Strand cDNA Synthesis Kit (Fermentas, Germany) using BioRad C1000 TouchTM Thermal Cycler (Bio-Rad Laboratories, USA). cDNA (20x diluted) was used as a template for qRT-PCR analysis performed using Maxima SYBR Green qPCR Master Mix (2×) (Fermentas, Germany) and Bio-Rad CFX96™ Real-Time PCR Detection system (Bio-Rad Laboratories, USA) in 20 μl PCR reaction mix. The PCR protocol consisted of 10 min 95°C initial denaturation, followed by 39 repeats of 30 s 95°C denaturation, 30 s 58°C annealing, 30 s 72°C extension and 5 s 73°C or 76°C and 80°C plate reading. Gene expression analysis was calculated by Bio-Rad Software Manager, Version 1.6, provided by the manufacturer (Bio-Rad Laboratories, USA). A normalized fold expression was compared with the ΔΔCq method. The target genes nuclear factor, erythroid 2-like 2 (NRF2) NFE2L2, heme oxygenase-1 (HO-1) HMOX1, catalytic subunit of glutamate-cysteine ligase (GCLC), NAD(P)H:quinone oxidoreductase 1 (NQO-1), as well as Kruppel-like factor 9 (KLF9) were normalized to the reference gene β-actin from each treated sample. Analysis was performed twice in triplicates. Obtained results were analyzed by T-test. The following primers were used: human β-actin (GI:2851): 5′-CCAACCGCGAGAAGATGACC-3′ (forward); 5′-AGGATCTTCATGAGGTAGTCAGTC-3′ (reverse); human NFE2L2 (GI:372620346): 5′-AGCAGGACATGGATTTGATTG-3′ (forward); 5′-TGGGAGA AATTCACCTGTCTC -3′ (reverse); human HMOX1 (GI: 49456890): 5′- ATGCCCCAGGATTTGTCA -3′ (forward); 5′- TGC AGCTCTTCTGGGAAGTA -3′ (reverse); human GCLC (GI: 308199421): 5′- GGCGATGAGGTGGAATACAT -3′(forward); 5′- GCATGTTGGCCTCAACTGTA -3′ (reverse); human NQO-1(GI: 1728): 5′-ACCTTGTGATATTCCAGTTCCCC-3′ (forward); 5′-TGGCAGCGTAAGTGTAAGCA-3′ (reverse); human KLF9 (GI:59853224): 5′-TCCGAAAAGAGGCA CAAGTG -3′(forward); 5′- CGTCTGAGCGGGAGAACTTT -3′ (reverse).

### Immunofluorescence

Cells grown on glass coverslips were fixed in ice-cold methanol. Non-specific binding was blocked by incubation with PBS containing 3% BSA for 60 min at 37°C. Cells were then incubated with primary antibody diluted in PBS with 1% BSA (PBS-BSA) for 1 h at 37°C. In these experiments, rabbit polyclonal antibody (1:500 dilution, Calbiochem, Merck Biosciences, Darmstadt, Germany) directed against 1829-1848 amino acid residues from human IP_3_R1 was used. Also, to determine ER marker calnexin, rabbit polyclonal antibody to synthetic peptide, corresponding to amino acids 50-68 of human calnexin (1:200 dilution, Abcam, Cambridge, UK) was used. This sequence is 100% conserved in human, mouse and rat IP_3_R1 protein. Afterwards, cells were washed three times with PBS-BSA for 10 min, incubated with CF Fluor® 488 goat anti-rabbit IgG (1:1000 dilution, Biotium, CA, USA) in PBS-BSA for 1 hour at 37°C and washed as previously. Finally, cells were mounted onto slides in mounting medium with Citifluor (Agar Scientific Ltd., Essex, UK), analyzed by laser scanning confocal microscopy (LSM 510 MetaMicroscope, Zeiss). Images were taken with Plan Neofluar 40x/1.3 oil objective. Images were scanned at scan speed 7 (260 Hz line frequency), 1024×1024 pixels, 12 Bit data depth in the average mode (4x line) at optical zoom 3. Z-stack interval was 0.8 micrometer. Images of all samples were acquired at the same microscope setup.

### *In vivo* experiments on nude mice

The animal experiment was carried out according to the recommendations of the European Community Guide for the Care and Use of Laboratory Animals and according to the experimental protocol approved by the Committee on the Protection of Animals, Faculty of Medicine, Masaryk University. Athymic nude mice (average weight 25.2 ± 2.2g) were housed under standard conditions. Tumor was induced by subcutaneous injection (5 × 10^6^ A2780 cells resuspended in PBS) into the area of scapula. Tumor was developed after 8-14 days. Size of the tumor was evaluated daily by caliper in two perpendicular directions.

Mice with the developed tumor were treated with SFN (40mg/kg), Xest (10pmol/g), combination of SFN and Xest, eventually by vehiculum (PBS). All compounds were applied daily, intraperitoneally during 7 consecutive days. Dose was calculated for each mouse according to the actual body weight. Status of each mouse was estimated each day. Tumor volumes were calculated according to the formula of the volume of an ellipse: V=4/3π x (a/2) x (b/2), where *a* and *b* corresponds to the two diameters of the tumor longest one and its perpendicular, respectively.

Day after the last dose the animals were sacrificed and metastatic dissemination was inspected. Primary tumor was explanted, weighed and documented.

### Statistical analysis

Each value represents an average of 3-9 wells from at least two independent cultivations of A2780 cells. Results are presented as mean ± S.E.M. Statistical differences among groups were determined by one-way analysis of variance. Statistical significance of at least p < 0.05 was considered to be significant. For multiple comparisons, an adjusted t test with p values corrected by the Bonferroni method was used (Instat, GraphPad Software).
